# Using Unplanned Fires to Help Suppressing Future Large Fires in Mediterranean Forests

**DOI:** 10.1371/journal.pone.0094906

**Published:** 2014-04-11

**Authors:** Adrián Regos, Núria Aquilué, Javier Retana, Miquel De Cáceres, Lluís Brotons

**Affiliations:** 1 CEMFOR-CTFC (Centre Tecnològic Forestal de Catalunya), Solsona, Spain; 2 CREAF (Centre de Recerca Ecològica i Aplicacions Forestals), Bellaterra, Spain; 3 Universitat Autònoma Barcelona, Bellaterra, Spain; University of Oxford, United Kingdom

## Abstract

Despite the huge resources invested in fire suppression, the impact of wildfires has considerably increased across the Mediterranean region since the second half of the 20^th^ century. Modulating fire suppression efforts in mild weather conditions is an appealing but hotly-debated strategy to use unplanned fires and associated fuel reduction to create opportunities for suppression of large fires in future adverse weather conditions. Using a spatially-explicit fire–succession model developed for Catalonia (Spain), we assessed this opportunistic policy by using two fire suppression strategies that reproduce how firefighters in extreme weather conditions exploit previous fire scars as firefighting opportunities. We designed scenarios by combining different levels of fire suppression efficiency and climatic severity for a 50-year period (2000–2050). An opportunistic fire suppression policy induced large-scale changes in fire regimes and decreased the area burnt under extreme climate conditions, but only accounted for up to 18–22% of the area to be burnt in reference scenarios. The area suppressed in adverse years tended to increase in scenarios with increasing amounts of area burnt during years dominated by mild weather. Climate change had counterintuitive effects on opportunistic fire suppression strategies. Climate warming increased the incidence of large fires under uncontrolled conditions but also indirectly increased opportunities for enhanced fire suppression. Therefore, to shift fire suppression opportunities from adverse to mild years, we would require a disproportionately large amount of area burnt in mild years. We conclude that the strategic planning of fire suppression resources has the potential to become an important cost-effective fuel-reduction strategy at large spatial scale. We do however suggest that this strategy should probably be accompanied by other fuel-reduction treatments applied at broad scales if large-scale changes in fire regimes are to be achieved, especially in the wider context of climate change.

## Introduction

Wildland fires are a major component of disturbance regimes in many regions [1]. While climate and vegetation characteristics have been described as major determinants of fire regimes, in the Mediterranean Basin and similar regions where human influence is widespread, fire regimes emerge as a complex process in which landscape planning, economic activities and fire management can override the influence of natural factors [2], [3], [4].

Despite the huge amount of resources invested in fire prevention and suppression, the impact of wildfires has considerably increased since the second half of 20^th^ century across different Mediterranean regions [1], [3], [5]. Fire suppression efforts have been stepped up in recent years, but while they appear to successfully deal with wildfires in mild weather conditions, there are doubts over the efficiency of these policies in climatically-adverse conditions [6], [7], [8]. Recent wildfires tend to be larger and more severe as a consequence of an increase in fuel accumulation and continuity (induced mainly by the abandonment of agriculture and livestock, and active afforestation policies) [3], [9], [10] coupled with drier and warmer climatic conditions [5]. In addition, the expansion of populations and the wildland–urban interface has also contributed to more fire ignition events [11].

In mesic regions of the Mediterranean basin (typically the Eastern Iberian Peninsula), fuel is now less limiting, and fire regimes appear to be mainly driven by the occurrence of climatically adverse conditions [12]. Different strategies can be envisaged to reduce the growing impact of wildfires in the Mediterranean region. Fire suppression policies have traditionally focussed on the preventive early detection of ignitions, but measures have recently shifted towards strategic planning and anticipation of fire spread to make optimal use of firefighting resources [13]. Other issues have been recognized as key, such as the management of fuel to reduce fire intensity and the extent and impact of large fires, but they pose difficulties in terms of effective implementation at large spatial scales [14], [15], [16], [17]. Fuel reduction may increase the chances of suppressing large fires in adverse climate conditions [8], [18]. Forest management (including grazing) has been proposed as a measure for reducing the accumulation of forest fuel. However, land abandonment is widespread in most regions affected by fire, and all available evidence suggests that forests are expanding in most of the Mediterranean [19], [20]. Prescribed fires are increasingly used to reduce fuel, but many countries face strong public opposition to this preventive action, making prescribed fires more difficult to apply at large scales for efficient fuel reduction than in other regions with Mediterranean-type climate, such as Australia [16], [21], [22].

Given that wildfires are currently seen as one of the main drivers of forest landscape changes in many Mediterranean regions, wildfires occurring in mild weather conditions could become a tool to regulate the impact of undesired, destructive, large fires taking place in climatically adverse conditions [6], [23]. Modulating fire suppression efforts in less adverse climatic conditions could allow a strategy to use unplanned fire events and the associated fuel reduction to create opportunities for efficient suppression of large fires in future adverse conditions [6], [24]. While such a strategy has the potential to control and reduce fuel using the current pattern of ignitions, any attempt to change the basic firefighting principle of tackling “all fires” as soon as possible would obviously meet with controversy, especially since very little information is available on the potential effectiveness of such a fire management strategy. Here, we address these questions using landscape simulations under different scenarios combining fire suppression strategies and climatic severity. Specifically, we investigate the potential of this opportunistic strategy for reducing the impact of large fires in climatically adverse conditions. We also assess what amount of area would need to be burnt in mild weather conditions to obtain reductions in the area burnt by large fires under a future climatic warming scenario. Finally, we discuss the spatial scale at which this strategy may be implemented, and its possible future socio-economic implications.

## Material and Methods

### Study area

**Figure 1:**
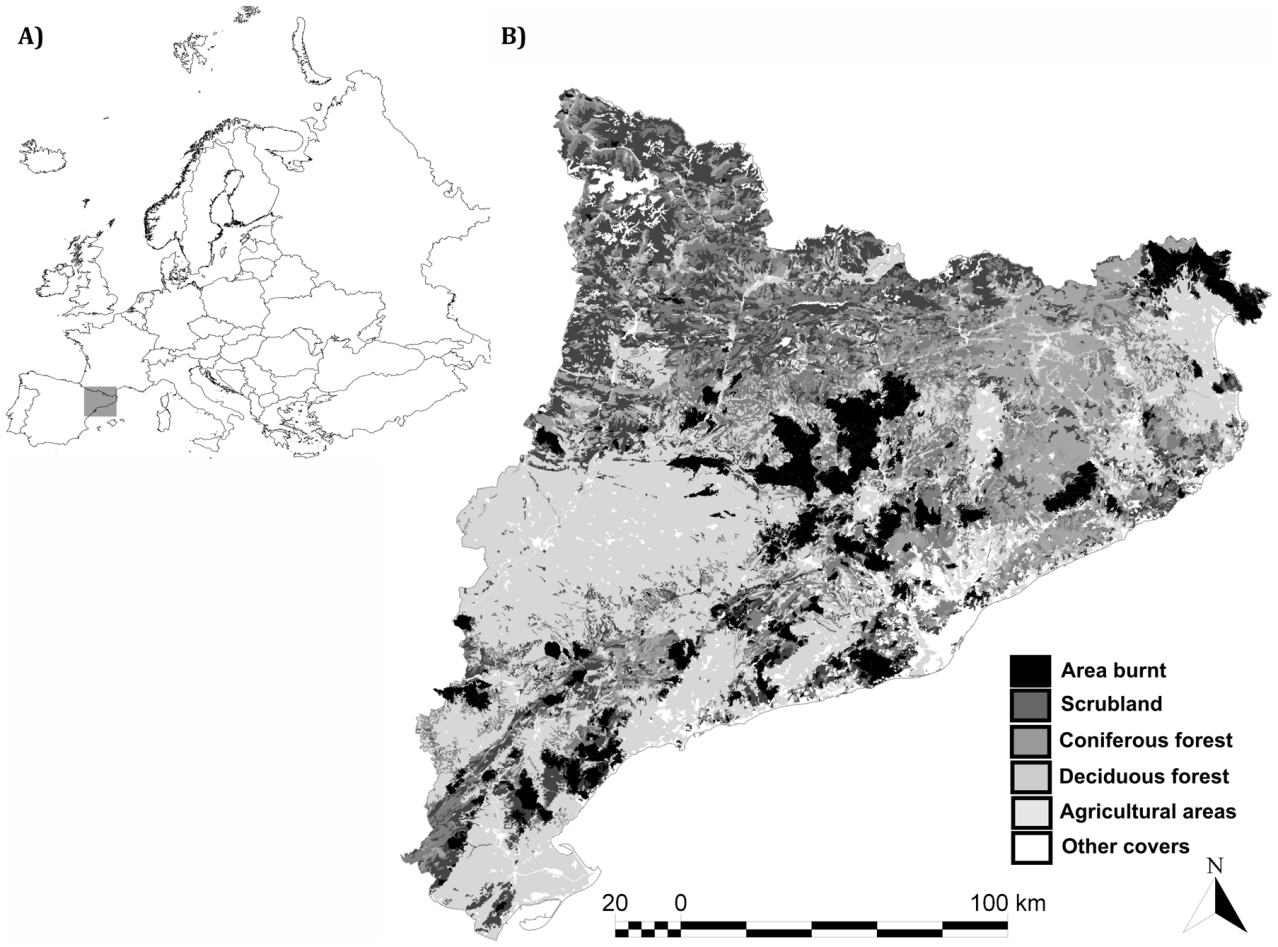
Location of the study area. Geographic location of the study area in south-western Europe (A). Land covers affected by fires between 1980 and 2000 in Catalonia (B).

The study area was Catalonia, a region located in northeastern of Spain with a land area of 32,115 km^2^ and an altitude that ranges from sea level to 3102 m. Catalonia has a typical Mediterranean climate with low winter precipitation and hot and dry summers. Moreover, its complex topography induces major variability in climatic and fire weather conditions across the territory. The vegetation is mostly comprised of forest and shrubland. Evergreen species occur in 60% of the total forest area, 73% of which is occupied by conifers (mainly *Pinus sylvestris*, *Pinus halepensis* and *Pinus nigra*), with sclerophyllous and deciduous species (*Quercus ilex*, *Quercus faginea* and *Quercus suber*) covering the remaining 40% [25]. According to the CORINE land cover map [26], shrubland of diverse species (and mainly evergreen) composition covers 37% of the total wildland area (Fig 1). Forest and shrubland were the most affected by fire during the 1975–1998 period [27].

During the same period, wildfires burnt about 13% of the wildland area (around 250,000 ha), and both the frequency of fire events and area burnt have increased since the pre-1970 period [12], [27]. This shift in fire regime was mainly driven by the increase in fuel amount and continuity following rural land abandonment across Catalonia during the pre-1970 period [10], [28]. As a consequence, resources allocated to fire suppression increased in the early 80s (*Focverd I* fire suppression program) and 90s (*Focverd II*) [29]. After 1999, fire-fighting capacities were improved through the creation of specific technical fire brigades (GRAF) whose mission is to understand fire behaviour and anticipate changes in fire propagation [29]. At the beginning of the 21^st^ century, prescribed burning programs were also implemented among other efforts to reduce fire risk, but not at sufficiently broad scales to achieve effective reductions [13]. Despite all these increased fire suppression and prevention efforts, wildfires continue to burn thousands of hectares in Catalonia every year. In addition, the aridity trends observed over recent decades point to an increase in the number of dry days per summer [5].

### MEDFIRE simulation model

The MEDFIRE model [4], [30] is a spatially-explicit landscape model that is able to mimic changes in landscape composition derived from vegetation dynamics and fire disturbances.

We present here a **short overview** of the model. The complete description, calibration, and validation processes for the study area can be obtained from previous published work [4]. The model was implemented using the version 3.5 of SELES modelling platform [31] (http://www.seles.info/). The current version of MEDFIRE (version 2) has two main sub-models [4]: (i) after-fire succession and maturation of vegetation (vegetation dynamics sub-model), and (ii) wildfire disturbance (fire sub-model) (more details in https://sites.google.com/site/medfireproject/). The main purpose of the model is to examine the spatial interaction between wildfires, vegetation dynamics and fire suppression strategies. It was designed to assess how different drivers affect fire regime at short- and medium-term timescales through a quantitative evaluation of their effects on the distribution of the annual area burnt, fire size distribution, and landscape composition. Validation exercises carried out for different time windows with different climate and fire suppression data showed that the model was able to reproduce the basic descriptors of fire regime in our study area [4].

The **state variables** that MEDFIRE uses to describe landscape context and conditions are spatially explicit variables in raster format at 100 m resolution. Land cover type (LCT) and time since last fire (TSF) are dynamic variables, while the static variables are: ignition probability, bioclimatic region, fire spread type (relief- or wind-driven), elevation, aspect and main wind direction.

The **fire sub-model** uses a top-down approach: for each time-step (a year), fires are simulated until the potential annual area to be burnt is reached. Potential annual area refers to the area that is expected to burn according to the historical fire data (1975–99 period). According to previous research [5], climatically adverse years are characterized by a high number of weather risk days (hereafter referred to as “adverse years”), as opposed to years dominated by mild weather conditions (hereafter called “mild years”). Thus, potential burnt area and fire size distributions depend on the climatic severity of the summer. For each simulated fire, the model chooses an ignition location used to establish the fire spread type [32]. The spread rate is a function of TSF (as a proxy of fuel accumulation), LCT flammability (of burnable land covers), aspect and wind direction (in wind-driven fires) or topography (in relief-driven fires). Fire spread rate was parameterized in a calibration exercise comparing model outputs with historical fire data [4]. In the absence of fire suppression, all the pixels that could be reached within the timespan of the fire are recorded as burnt (i.e. post-fire transitions may occur, and the TSF is set to 0). Otherwise, if fire suppression occurs, the pixels recorded as burnt include only a subset of pixels that were not affected by fire suppression, and therefore the final fire size will be smaller than was potential. We assume that long-term droughts or long periods with high temperatures or strong winds can be predicted and, therefore, high-risk fire conditions can be anticipated before an ignition takes places thus permitting firefighters to distinguish fire conditions. Thus, although the model assumes that climate is the main driver of fire regime, key elements of fire regime such as fire size can be modulated by fire suppression.

The **vegetation dynamics sub-model** assumes that forest cover types are relatively stable, so a type-conversion can only occur after burning. Succession without burning can occur only from shrubland to forest. This land cover change takes place depending on the availability of mature forest in neighbouring cells and the TSF of shrubland that will potentially change. Once a cell is burnt, this sub-model updates the land cover according to two post-fire regeneration approaches:

- Applying non-spatial stochastic transitions, using a multinomial distribution with transition probabilities previously published by others [33] that depend on pre-fire cover class as well as on other factors such as aspect, bioclimatic region and TSF.
- By neighbourhood species contagion, considering the neighbours that were also burnt in the current year and shared the same pre-fire cover class. It is important to note that the model cannot handle the complexity derived from possible within-stand heterogeneity since each cell can only be described by a single dominant tree species.

### Fire suppression strategies in MEDFIRE

**Figure 2:**
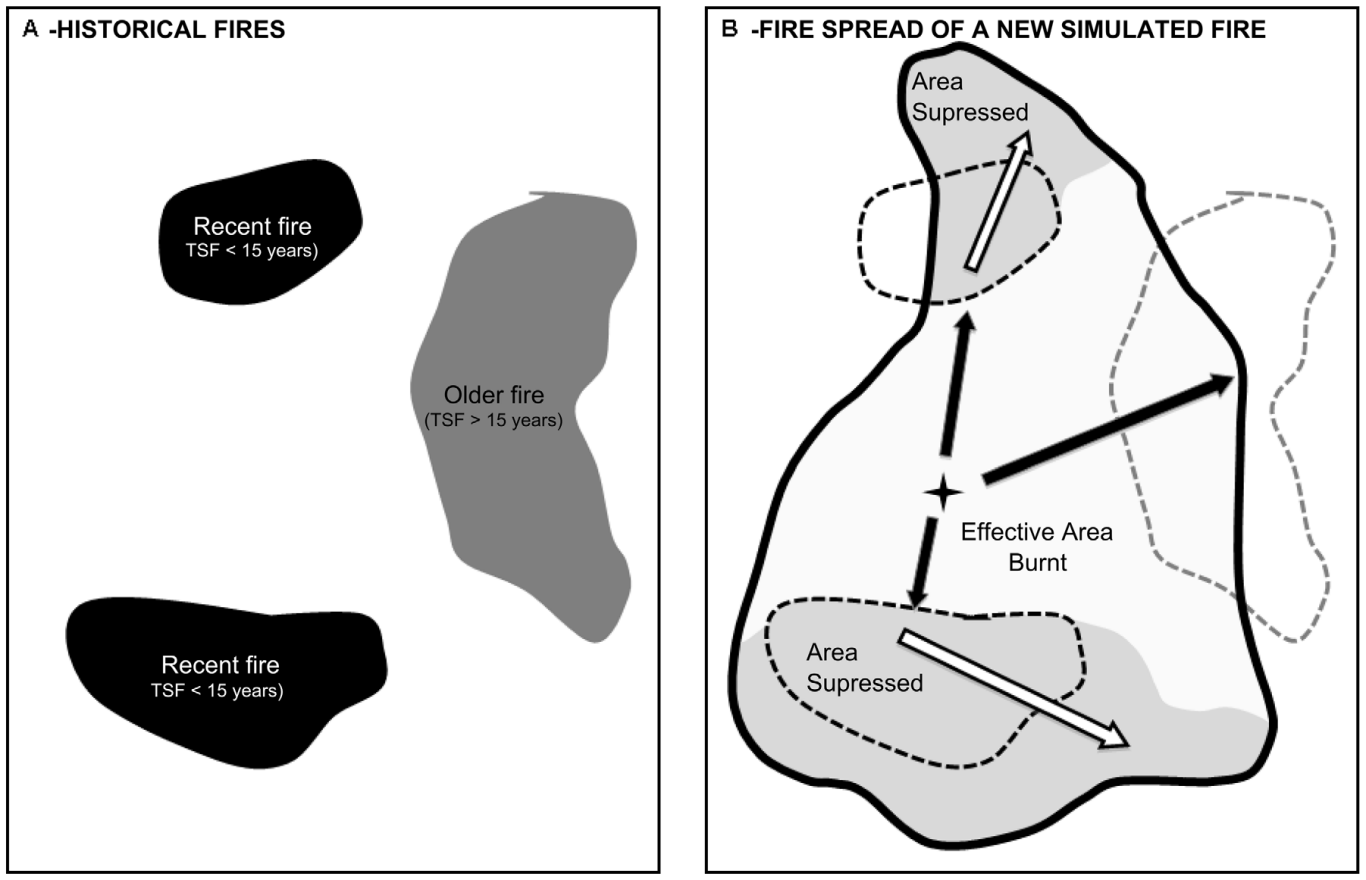
The figure, modified from previous work [4], illustrates the effects of opportunistic fire suppression on realized area burnt. (A) Historical fires in a region, where black patches are recent fire scars with time since last fire less than 15 years and grey patches correspond to older fire scars. (B) Fire spread of a new simulated fire in the area. Potential area (thick black line) is larger than the final area burnt due to opportunistic fire suppression generated by recent fires in (A). Suppressed areas are shown in grey and main spread axes are arrowed. Spread occurring within final area burnt (black arrows) and potentially within the suppressed area (white arrows) is shown.

Two fire suppression strategies are implemented in the MEDFIRE model:

- **The opportunistic strategy** mimics firefighting actions based on the ability to take advantage of opportunities derived from old fire scars. These fires provide firefighting opportunities since they are easy to detect by fire brigades and they strongly decrease fuel load and therefore fire intensity. The implementation of the opportunistic strategy in MEDFIRE suppresses burning in a cell whenever its TSF is below a pre-specified threshold (expressed in years) (Fig. 2).
- **The active fire suppression strategy** mimics the overall efficiency of firefighters to anticipate fire behaviour and reduce realized area burnt under determinate fire propagation conditions. This strategy was implemented through two different processes. First, we induced increases in the potential annual area to let burn so as to reproduce the effects of not suppressing small fires and therefore increase the total number of fires in a given scenario. By default, the MEDFIRE model simulates fires until the potential annual area to be burnt is reached, and so increases in potential area burnt result in a larger number of fires per year. Second, we introduced the concept of opportunities tied to fire-specific thresholds in spread rate. In areas in which spread rate is below a pre-specific threshold, firefighters are able to stop the fire spreading, which leads to decreases in the final area burnt [4].

### Scenario definition

**Figure 3:**
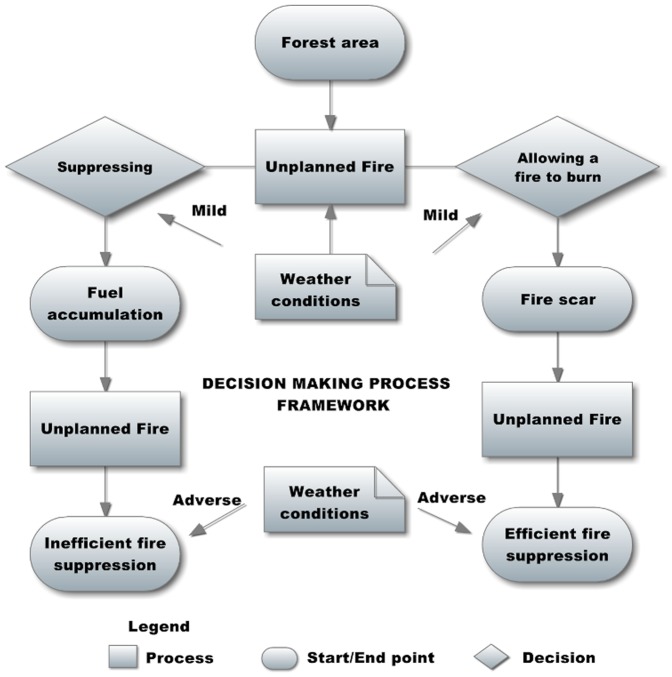
Decision-making process for an opportunistic fire suppression policy based on whether or not unplanned fires should be let to burn in mild weather conditions.

We assessed the effectiveness of an opportunistic fire suppression policy based on whether or not to allow unplanned fires to burn in mild weather conditions through scenarios characterized by a progressively decreasing active fire suppression policy (Fig. 3). Specifically, we designed eighteen future scenarios by combining different fire suppression strategies and levels of climatic severity (Tables 1 and 2). We considered **nine different treatments of active fire suppression** (acting only in years with mild climatic conditions) corresponding to situations of variability in general fire suppression efforts. The nine treatments were defined by combining three levels of potential area to be burnt with three levels of active suppression using spread rate thresholds: (a) the first three levels were simulated through variability in potential area to burn. Baseline annual distributions of area burnt were derived from 1975-99 wildfire statistics (using a lognormal distribution fitted for the available data). To reproduce the effects of not suppressing small fires (leading to increases in the total area burnt), we modified the mean of the lognormal distribution of area burnt in mild years according to the decreasing efforts of firefighters to suppress small fires: (1) *High* 7.74 (∼6,500 ha/year); (2) *Average* 9.14 (∼26,000 ha/year); and (3) *Low* 9.81 (∼52,000 ha/year) (Tables 1 and 2). (b) The second three levels were defined using specific spread rate thresholds to reduce the final area burnt: (1) strong active suppression of opportunities corresponding to spread rate of heading fires, or descending fronts in pine forests (fire spread threshold 90); (2) medium active suppression of opportunities corresponding to spread rate in agricultural cover and sclerophyllous forest (fire spread threshold 40); (3) no active suppression of opportunities (fire spread threshold 0) (Tables 1 and 2). **Opportunistic fire suppression** was only allowed in climatically adverse years and was characterized by the number of years since the last fire in which the fire scars can be used as fire suppression opportunities by firefighters. In all simulation scenarios, the opportunistic fire suppression strategy was limited to fire scars generated in the last 15 years. The ongoing climatic trends show an increase in the number of years with very high fire-risk days [5]. Finally, we used **two climatic treatments** describing whether the percentage of adverse years (*i.e.* with dry and hot summers) will remain stable in the future (35% is the percentage of adverse years in the period 1980–1999) or is set to increase (up to 70%), following recent research [4] (Tables 1 and 2). Five hundred replicates of each scenario were simulated for a 50-year period (2000–2050).

**Table 1:** Description of the variables and levels used in the scenarios characterization.

Variables	Levels	Description
Active fire suppression	90	Strong active suppression corresponding to spread rate of heading fires or descending fronts in pine forests
	40	Medium active suppression corresponding to spread rate in agricultural cover and sclerophyllous forest
	0	No active suppression
Potential area to burn	6,500 (high)	Hectares/year to burn in climatically mild years according to historical fire statistics (1975–1999). Period characterized by strong efforts of firefighters to suppress small fires (*Focverd I* and *II* fire suppression programs)
	26,000 (average)	Hectares/year to burn in mild years considering an average efforts of firefighters to suppress of small fires
	52,000 (low)	Hectares/year to burn in mild years considering relatively little effort of firefighters to suppress small fires
Opportunistic fire suppression	15	Number of years since the last fire after which fire scars can be used as fire suppression opportunities by fire brigades
Climatic severity	35	Percentage of adverse years for the simulation period (2000–2050) according to the trends recorded in the period 1980–1999
	70	Percentage of adverse years for the simulation period (2000–2050) factoring in climatic warming

**Table 2:** List of simulation scenarios describing parameters used to reproduce active firefighting strategies and climate severity.

ID	Factors		
	Active Fire Suppression		Climatic Severity
	Potential annual area to burn (ha/year)	Spread rate threshold	Adverse years (%)
	*Mild years*	*Mild years*	
1	6,500	90	35
2	6,500	40	35
3	6,500	0	35
4	6,500	90	70
5	6,500	40	70
6	6,500	0	70
7	26,000	90	35
8	26,000	40	35
9	26,000	0	35
10	26,000	90	70
11	26,000	40	70
12	26,000	0	70
13	52,000	90	35
14	52,000	40	35
15	52,000	0	35
16	52,000	90	70
17	52,000	40	70
18	52,000	0	70

Potential annual area to burn follows a lognormal distribution (with mean 7.74 and standard deviation 1.43 in mild years fitted from 1975–99 wildfire statistics). The mean of that distribution is used as a scenario parameter with three possible values: (1) **High**-7.74 (∼6,500 ha/year), (2) **Average**-9.14 (∼26,000 ha/year), and (3) **Low**-9.81 (∼52,000 ha/year).

### Evaluation of simulation results

To evaluate the effect of opportunistic fire suppression strategies under the different scenarios, we calculated area suppressed as the difference between potential area to be burnt in a year and the final area burnt. We also tracked the area suppressed in adverse years according to the origin of the fire scar that created the firefighting opportunity: (1) scars of fires simulated in climatically adverse years, (2) scars of fires simulated in climatically mild years, and (3) scars of historical fires (those affecting the region before the simulation started, i.e. the 1975–99 period). The means and standard deviations of all these variables were used to describe the fire regime obtained under each simulated scenario. All statistical analyses were performed using R software, version 3.0.2 [34].

## Results

### Effects of decreasing fire suppression in mild weather on later undesired large fires

**Figure 4:**
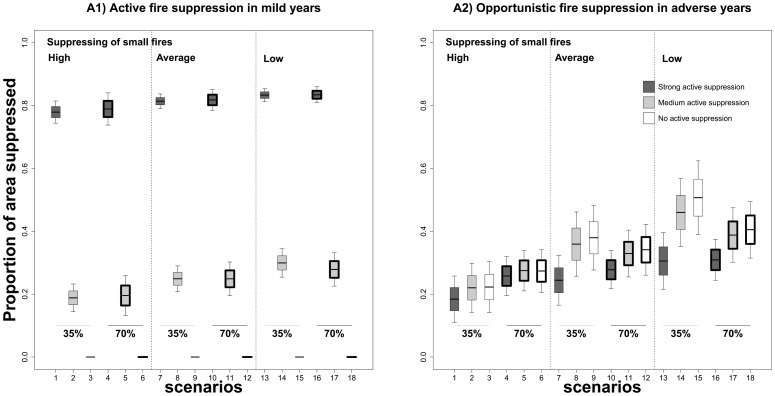
Proportion of area suppressed in relation to potential area to burn. Area suppressed by active fire suppression strategies in mild years (A1) and opportunistic fire suppression strategy in adverse years (A2). Potential annual area in mild years follows a lognormal distribution with means of 7.74 (**High** ∼6,500 ha/year), 9.14 (**Average** ∼26,000 ha/year), and 9.81 (**Low** ∼52,000 ha/year), to reproduce a decreasing effectiveness of firefighter efforts to suppress small fires. Simulation scenarios characterized by thresholds of 90% in active fire suppression of mild years are represented in dark-grey box-plots, in light grey by thresholds of 40%, and in white of 0%. Box-plot elements are as follows ― lower and upper whiskers: approximately 68% of all data values (**mean ± SD**, standard deviation); lower and upper midhinges: the **mean ± ½ SD**; central black line: the **mean**. Box outline width represents the percentage of adverse years used in the simulations, i.e. 35% (thin width) and 70% (thick width).

**Figure 5:**
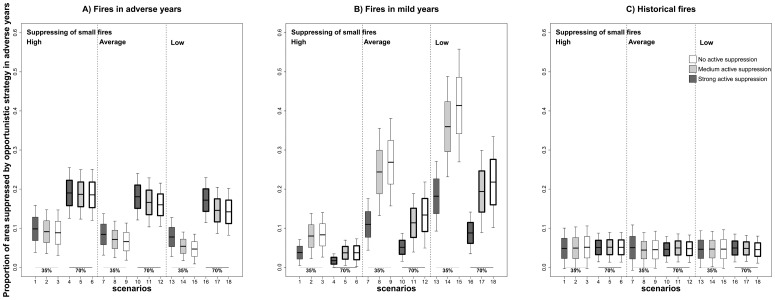
Proportion of area suppressed (in relation to potential annual area) by opportunistic fire suppression strategy in adverse years. Area suppressed by firefighting opportunities derived from: (A) fires in adverse years, (B) fires in mild years, (C) historical fires. Simulation scenarios characterised by thresholds of 90% in active fire suppression of mild years are represented in dark-grey box-plots, in light-grey by thresholds of 40%, and in white of 0%. The labels *high*, *average* and *low* refer to the distribution of potential annual area in mild years (see Fig 4 for more details). Box-plot elements and box-plot outline width are as in Fig. 4.

Scenarios with no and medium active fire suppression in mild years (see scenarios charted with white and light-grey box-plots in Fig. 4, A2) showed an increase in fire suppression opportunities in adverse years compared to scenarios characterized by strong fire suppression (see dark grey box-plots in Fig. 4, A2). Specifically, the area suppressed in adverse years by opportunistic strategies increased from 18% to 29% in scenarios with strong active fire suppression (dark-grey box-plots in Fig. 4, A2), and from 22% to 50% with no active firefighting in mild years (white box-plots in Fig. 4, A2). Moreover, the opportunities derived from historic fire scars were the same in all scenarios (Fig. 5C), showing that the increased efficiency of the opportunistic strategy came from simulated fires.

Looking at the amount of area that would need to be burnt per year to reduce the impact of large fires in extreme fire weather, results show that the reference scenarios built on historical wildfire statistics (scenarios labelled *high* from 1 to 3, burning approximately 6,500 ha/year in mild years) allowed opportunistic suppression of 18–22% of the target annual area in adverse years (Fig. 4, A2). If mean area burnt in mild years was increased to 26,000 ha/year by decreasing efforts to suppress small fires (scenarios labelled *average* from 7 to 9), the area suppressed in adverse years would increase to 26–37% (Fig. 4, A2). Finally, when mean area burnt in mild years was increased to 52,000 ha/year due to a fire suppression policy aimed at engineering a shift in fire regime towards a higher number of small fires (scenarios labelled *low* from 13 to 15), the percentage of area suppressed in adverse years also increased to figures of up to 29–50% (Fig. 4, A2). However, while the opportunities derived from fires simulated in mild years (see scenarios 1–3 compared to scenarios 7–9 and 13–15 in Fig. 5, B) increased as the final annual area burnt in these years grew, the opportunities generated in earlier adverse years decreased (see scenarios 1–3 compared to scenarios 7–9 and 13–15 in Fig. 5, A). This increase in fire suppression opportunities was therefore partially modulated by the decrease in opportunities from adverse years.

### Interaction between fire suppression and climatic severity

To evaluate the interaction between fire regime, fire suppression and climate warming, we compared scenarios with the same set of parameters while varying the percentage of adverse years from 35% up to 70% (see Table 1). When the number of years with adverse conditions increased, counterintuitive effects appeared on the potential opportunistic fire suppression strategies. Thus, the area suppressed was higher in the simulated scenarios under climate warming (scenarios 4–6 in Fig. 4, A2) than under the current climatic regime (scenarios 1–3 in Fig. 4, A2). Climate warming induced a greater area suppressed by opportunities derived from fires simulated in adverse years (compare scenarios under climate warming, boxed in a thick line, and scenarios without climate warming, boxed in a thin line, in Fig. 5, A). However, when we considered a more severe fire regime in mild years (∼52,000 ha/year, scenarios with label *low*), the area suppressed was lower in scenarios with climate warming (scenarios 10–12 and 16–18 in Fig. 4, A2) than scenarios without climate warming (scenarios 7–9 and 13–15 in Fig. 4, A2). This increase in area burnt in mild years again led to a greater area suppressed by opportunities derived from fires simulated under mild weather conditions (compare scenarios labelled *high* and *low* in Fig. 5, B). However, it is also remarkable that climate change also led to fewer years with mild fire weather conditions, thus reducing the window of opportunity for the creation of opportunities under these conditions (compare scenarios with climate change, boxed in a thick line, against scenarios without climate warming, boxed in a thin line, in Fig. 5, B).

## Discussion

We have shown that relaxing fire suppression efforts under relatively controlled conditions (opportunistic fire suppression policy) has the potential to substantially reshape fire regimes and decrease the amount of area burnt under undesired, extreme climate conditions. However, the potential of this strategy is somewhat limited due to the complexity of the interactions between fuel availability, fire impact, and fire suppression strategies.

### Potential of opportunistic strategy for reducing the impact of large fires

Given the growing impact of wildfires, the effect of fire exclusion has been extensively studied and debated in many different Mediterranean ecosystems for decades now [2], [35], [36]. Many authors claim that the systematic extinction of “all fires” leaves an accumulation of fuel that will be consumed in future large fires in years with extreme fire weather conditions (fire paradox) [35], [37], [38]. Recent studies carried out in the Mediterranean basin using fire-succession models have demonstrated that high fire suppression efforts may lead to a slightly higher proportion of large and more intense fires [2], [39]. Our results concur with this hypothesis, since scenarios with strong active fire suppression in mild weather did lead to increases in the area burnt by large fires in adverse conditions due to feedbacks in the dynamics of fuel accumulation (Fig. 4). In addition, we demonstrated that relaxing the fire suppression efforts in mild years, in which benign fire weather conditions allow firefighters to tackle any fire event efficiently, provided additional fire scars associated to fuel reduction. These new burnt-area patches generated potential firefighting opportunities for later adverse years (Fig. 5, B). In our particular case, the area suppressed in adverse years by opportunities derived from fires simulated in mild years increased considerably, as the final annual area burnt in these years was higher (Fig. 5, B). In the reference scenarios derived from statistics on wildfires between 1975 and 1999 (which burnt 6,500 ha/year in mild years), the area suppressed by opportunities derived from previous fire scars only accounted for 22% of the potential annual area in adverse years (Fig. 4). This suggests that the current high-efficiency fire suppression policy may be decreasing the opportunities that arise from past fires. Our results showed that a progressive increase of area burnt in mild years by relaxing fire suppression efforts led to a reduction in area burnt in adverse years: a decrease of an additional 15% (up to 37%) would require a four-fold increase in the final annual area burnt in mild years (i.e. to 26,000 ha/year), whereas a decrease of an additional 23% (up to 50%) would require an eight-fold increase (i.e. to 52,000 ha/year). Therefore, to effectively reduce large fires under adverse weather conditions, we need to allow burning across larger areas in mild years. Bear in mind that reductions in the total area burnt in adverse weather conditions do not come for free ― here, the reductions involved a loss of future opportunities for firefighting (Fig. 5, A) [4]. Therefore, the amount of area treated in mild conditions has to be very high to reduce the amount of area burnt in adverse climate conditions and compensate for the loss of opportunities derived from fires avoided in such adverse conditions.

Fire management based on climate-adapted modulation of fire suppression shares some overlap with the management policy of prescribed burnings implemented in other Mediterranean ecosystems [15], [21], [22]. Both prescribed burning and the use of unplanned fires resulting from decreasing suppression efforts are tactics that use fire as a tool to fight larger wildfires and that aim to increase the effectiveness of fire suppression through fuel reduction [8], [18]. Recent studies [2], [39] used a simulation model to investigate whether large fires in the Mediterranean region are consequence of large fire suppression programs or, conversely, are driven by extreme fire weather conditions. Their results suggest that, although the total area burnt is much the same regardless of whether or not fire suppression or prescribed fire policies are used, prescribed burning does reduce fire intensity. Here, we show that like prescribed burning, unplanned fire events could be used to reduce fuel accumulation and fire intensity to create opportunities for effective fire suppression of large fires in future adverse conditions. We therefore suggest that designing treatments to minimize adverse fire effects may be a more effective strategy than designing treatments that attempt to extinguish “all fires”.

Moreover, in our study and in the current fire regime context, unplanned fires increase landscape heterogeneity but do not seem enough to offset the decade-long general trend towards homogenization due to land abandonment and the coalescence of natural vegetation patches [28], [40]. This landscape homogenization process is driving an increase in fuel continuity [41] and consequently fire spread and intensity [10], [12], [42]. To offset this ongoing trend and create new fire suppression opportunities, we envisaged a fuel-reduction strategy based on relaxing fire suppression efforts in mild years to create a novel fire regime with a large number of smaller fires. Our results have demonstrated that this strategy is associated with effective reductions in the area burnt by fires in adverse years. Thus, decreasing fire suppression in mild weather conditions may create landscapes in which wildfires occur with less devastating consequences. Fire may itself play a key role in maintaining these novel landscapes.

### Opportunistic fire suppression under climatic warming scenarios

The interaction between area burnt, fire suppression and climate warming had counterintuitive effects on the potential for opportunistic fire suppression strategies to reduce the amount of area burnt in climatically adverse years. The area burnt in adverse years in scenarios with climate warming is consistent with climate change bringing warmer and drier summers and increased fire weather risk [43], [44]. Overall, our results are in agreement with the trends reported for recent decades in the Mediterranean basin [5], [45]. However, the area burnt in the simulated scenarios under climate warming was lower than expected due to increases in the area suppressed by opportunities derived from simulated fires in adverse years (Fig. 5, A). At the same time, climate warming also implies a lower number of mild years and therefore fewer windows for creating the opportunities targeted. In this context, exponentially larger areas need to be burnt in mild years to create additional fire suppression opportunities (Fig. 5, B). On the other hand, recent studies carried out in regions with Mediterranean climatic conditions highlight the role of landscape structure in shaping current and future fire–climate relationships at regional scale, and suggest that the future changes in fire regime under global warming may be different from what it is predicted by climate alone [46]. We argue that opportunistic fire suppression policies have the potential to substantially effect changes in this fire–climate relationship through the novel landscapes created by relaxing active fire suppression efforts in mild weather conditions. We also suggest that, given the large annual area burnt required to prevent large fires in adverse fire weather years, the spatial allocation of firefighting efforts and fire suppression resources will be a keystone for the optimization of this fuel-reduction strategy and its successful implementation in future firefighting programs forced to deal with climate change.

### Implications for the future: some considerations

The effectiveness of this opportunistic fire suppression strategy is still relatively low compared to other fuel reduction strategies applied in different Mediterranean-type ecosystems [15], [18], [47]. In Australia, where prescribed burning is used as a cost-effective fuel-reduction treatment, previous works [22] found that three units of prescribed fires were required to reduce one unit of unplanned fire area. This negative relationship was stronger in the tropical savannas of northern Australia, where prescribed early-dry-season burning was able to substantially reduce late-dry-season fire area by direct one-to-one replacement [16]. In Australian eucalypt forest, other authors [21] found that each unit area reduction in unplanned fire required about four units of prescribed fire. We argue that strategic placement of these fuel treatments (*i.e.* prescribed burning or thinning followed by prescribed burning) is the likely key to effective implementation. In fact, recent research [48] found that strategic placement of fuel treatments reduced the predicted growth rates of simulated fires under adverse weather conditions more effectively than random placement in three study areas of western USA. Random placement of fuel treatments required about twice the treatment rate of optimally-placed fuel treatments to yield the same reduction in predicted fire growth rates [48]. However, opportunistic fire suppression is based on unplanned fire occurrence. Unplanned fires tend to be determined by the spatial arrangement of ignition factors. In Mediterranean countries where natural ignitions are scarce, fire regime is strongly linked to human activities, with the result that fire scars are not randomly distributed in the space but follow the auto-correlated pattern of human activities [1], [49], [50]. Therefore, we suggest that the identification of spatio-temporal patterns of fire occurrence at regional scales in these systems may optimize the opportunities created by unplanned fires, and thus mitigate the heavy impact of the undesired large fires in extreme fire weather.

From a purely economic standpoint, opportunistic ‘let-burn’ fire suppression strategies have a further benefit tied to the fact that they curb the economic losses caused by large fires while also saving on fire suppression resources. In fact, a recent study suggests that the potential savings associated to opportunistic strategies could be substantial [23]. The authors simulated unplanned fires at landscape scale over a 100-year period using existing models of fire behaviour, vegetation and fuel development and fire suppression effectiveness to estimate suppression costs using a suppression cost model. They found that estimated future suppression cost savings were positively correlated with fire size. Others authors [51] studied different spatial factors influencing large wildland fire suppression expenditures, and they also found that fire size and private land had a strong effect on expenditures. Note that just a tiny fraction of fires (around 1%) accounts for 85% of suppression expenditure in the western USA [6]. Given that the land in our study area is mainly privately-owned, we suggest that an opportunistic strategy aimed at mitigating large wildfires by relaxing efforts to suppress small fires could enable huge suppression savings. However, this strategy also implies large areas burnt in mild weather conditions. The decision on whether a particular fire should be let to burn must be made by weighing up the potential benefits in terms of the landowners' management objectives and the potential cost of damage from unsuppressed fire [52]. It is the net benefit of allowing a fire to burn that is the relevant criterion [23], [53]. Therefore, factoring in the potential socio-economic and ecological costs (such as soil loss, destruction of wildlife habitat, loss of timber value, infrastructure and human life) and benefits discussed here is essential for identifying candidate areas suitable for using unplanned fires as a management tool.

To conclude, we suggest that to achieve the stand structure and fuel reduction goals required to minimize large fires in extreme fire weather, this strategy could be accompanied by other fuel-reduction treatments such as large-scale forest thinning or biomass extraction. Further studies are needed to assess the impact of novel fuel-reduction treatments on fire regime. Moreover, the possible impacts of these fire management options on biodiversity and a variety of ecosystem services should be carefully evaluated before cost–benefit analyses can be developed. These potential fuel-reduction treatments should therefore also be evaluated in terms of social, economic and environmental cost–benefit trade-offs.
